# Publication Bias in Psychology: A Diagnosis Based on the Correlation between Effect Size and Sample Size

**DOI:** 10.1371/journal.pone.0105825

**Published:** 2014-09-05

**Authors:** Anton Kühberger, Astrid Fritz, Thomas Scherndl

**Affiliations:** 1 Department of Psychology, University of Salzburg, Salzburg, Austria; 2 Centre for Cognitive Neuroscience, University of Salzburg, Salzburg, Austria; 3 Österreichisches Zentrum für Begabtenförderung und Begabungsforschung, Salzburg, Austria; Université de Montréal, Canada

## Abstract

**Background:**

The *p* value obtained from a significance test provides no information about the magnitude or importance of the underlying phenomenon. Therefore, additional reporting of effect size is often recommended. Effect sizes are theoretically independent from sample size. Yet this may not hold true empirically: non-independence could indicate publication bias.

**Methods:**

We investigate whether effect size is independent from sample size in psychological research. We randomly sampled 1,000 psychological articles from all areas of psychological research. We extracted *p* values, effect sizes, and sample sizes of all empirical papers, and calculated the correlation between effect size and sample size, and investigated the distribution of *p* values.

**Results:**

We found a negative correlation of r = −.45 [95% CI: −.53; −.35] between effect size and sample size. In addition, we found an inordinately high number of *p* values just passing the boundary of significance. Additional data showed that neither implicit nor explicit power analysis could account for this pattern of findings.

**Conclusion:**

The negative correlation between effect size and samples size, and the biased distribution of *p* values indicate pervasive publication bias in the entire field of psychology.

## Introduction

Theories are evaluated by data. However, since whole populations cannot be examined, statistics based on samples are used for drawing conclusions about populations. The *p* value of a significance test is the main statistic used for making such inferences. Yet the use the *p* value is often criticized [1], [2], since *p* values lead to dichotomous reject/not reject decisions, and to the misconception that significance means a large effect while non-significance means no effect [3], [4]. It has therefore been recommended that estimates of effect size (ES) should accompany *p* values [5]–[7]. Reporting of ES has increased recently [8], but ES are still widely neglected [9].

### The Relationship between Effect Size and Sample Size

An ES is a measure of the strength of a phenomenon which estimates the magnitude of a relationship. Thus, ES offer information beyond p-values. Importantly, ES and sample size (SS) ought to be unrelated. Here we provide evidence that there is a considerable correlation between ES and SS across the entire discipline of psychology: small sample studies often produce larger ES than studies using large samples. Publication bias can be a reason for a correlation between ES and SS (as we will argue here), but there can be other reasons: power analysis, the use of multiple items, and adaptive sampling.

Power analysis. Statistical power is the probability of detecting an effect in a sample if the effect exists in reality. Power analysis consists in choosing SS in a way to ensure high chances to detect the phenomenon given the anticipated size of the effect. Thus, if we expect a large ES, power analysis will show that a small SS is sufficient to detect it. In contrast, if we expect a small effect, power analysis calls for a large SS. This procedure of SS determination leads to a negative relationship between ES and SS.

Repeated trials, or multiple items. Overestimations of ES can result from aggregating data [10]. This occurs because, as the number of trials increases, the pooled standard deviation decreases. ES is computed as the difference between groups divided by the pooled standard deviation, and ES will therefore increase.

Adaptive sampling. Lecoutre et al. [11] showed that researchers collect more data if the results are nearly, but not quite, statistically significant, and John et al. [12] estimate the prevalence of researchers having ever engaged in adaptive sampling to be near 100%. Yet, any stopping rule that takes prior results from a significance test into account (in an extreme case, stop if significant and continue if non-significant) will lead to a negative ES - SS correlation.

Besides these three main arguments for a correlation between ES and SS there are additional possibilities which can result in such a correlation. First, it might be that smaller experiments are conducted in more controlled (lab) settings, or that smaller experiments use more homogeneous samples (e.g., psychology undergraduates) than larger experiments (e.g., ran via Amazon's Mechanical Turk). Yet, empirical evidence does not fully support this view as there are strikingly similar results from MTurk as well as lab-experiments [13]. Second, in clinical work, smaller samples could include the more severely afflicted cases than larger samples. Third, larger studies can be multi-factorial, including crossed factors that are aimed to study moderation of the effect, making the ES smaller than in the smaller studies which tend to be uni-factorial. This argument rests on the assumption that moderation effects are generally tested with bigger samples. This is plausible, but not fully consistent with the long known problem that moderation effects are studied with strongly underpowered samples and evidence that sample size of moderation studies are not much larger than ‘typical’ sample size [14]. Fourth, it has been argued that authors could act strategically and set up smaller experiments in order to have more maneuverability when analyzing a set of experiments [15]. Finally, in some lines of research there may be substantive reason for funnel plot asymmetry. For instance, in social psychological studies of (implicit or perceived) discrimination those who are in a more apparent minority position (e.g., minority students at an Ivy League school) may be relatively more susceptible to certain effects, which could also show up as genuine relation between ES and SS.

All these explanations may well be true to some extent. However, in most cases when funnel plot asymmetry is found, it is attributed to publication bias [16], [17], We agree: The common ground for a correlation between ES and SS is – directly or indirectly – publication bias. Publication bias is present if significant results have a better chance of being published. Publication bias can occur at any stage of the publication process where a decision is made [18], [19]: in the researcher's decision to write up a manuscript [20]; in the decision to submit the manuscript to a journal [21], [22]); in the decision of journal editors to send a paper out for review [23]; in the reviewer's recommendations of acceptance or rejection [24], and in the final decision whether to accept the paper [25]. Anticipation of publication bias may make researchers conduct studies and analyze results in ways that increase the probability of getting a significant result [26], and to minimize the danger of non-significant results [27].

Publication bias leads to a negative ES-SS correlation, which has been reported in some research areas [28]–[33]. However, there could also be publication bias in studies on publication bias (although Dubben & Beck-Bornholdt [34] find no statistical evidence for this), and therefore the picture might be incomplete. Our question thus is: Is this a problem for some restricted research areas, or for the entire discipline of psychology? If so, how strong is the relationship, and what does it mean for psychology as a science?

## Method

### Sampling, Classification, and Analysis

**Figure 1:**
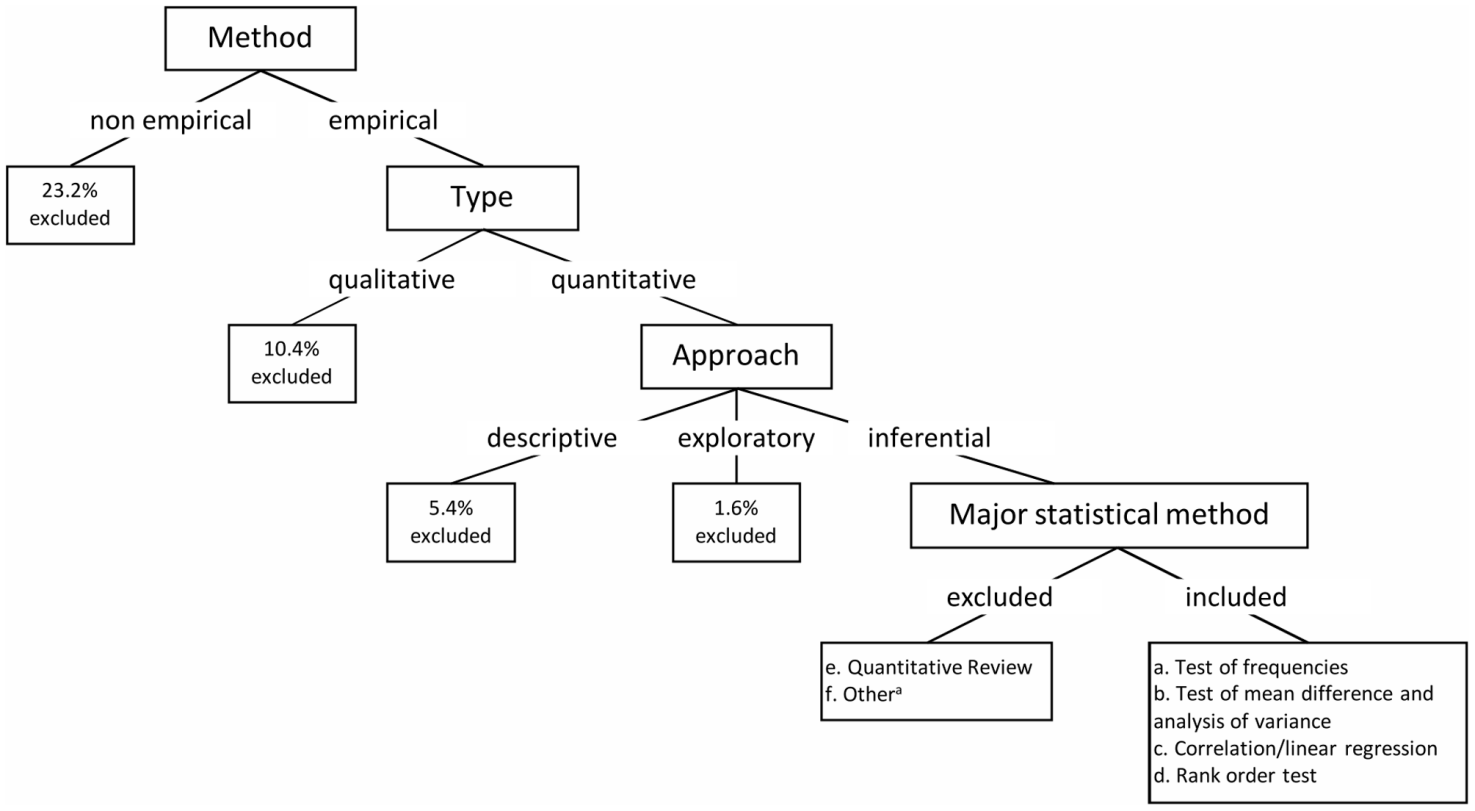
Decision tree for classification of articles. Note: aConfirmatory factor analysis; path analysis; structural equation modeling; hierarchical linear modeling; survival analysis; growth curves; analyses testing reliability and validity of scales.

A random sample of 1000 English-language peer reviewed articles published in 2007 was drawn from the PsycINFO database. As keywords we used ‘English’ ‘peer reviewed’ ‘journal article’ in ‘year 2007’. Three articles could not be acquired, another article was a duplicate. The remaining 996 articles were classified using a hierarchical tree (see Figure 1). Of the 996 articles, roughly one fourth (23.2%) were not empirical. The 765 empirical articles were further classified according to basic methodology: qualitative (13.6%) and quantitative. Papers were coded as qualitative when the analysis was done by coding and categorizing of interviews, pictures, videos, or similar data without using inferential statistics. The 661 quantitative articles were classified according to purpose: descriptive (8.0%), exploratory (2.4%), or inferential (89.6%). An article was coded descriptive if data were summarized quantitatively without inferring population values by statistical means; the latter were coded inferential. Exploratory articles used some ‘structure detecting’ method (e.g., principal component analysis, factor analysis, cluster analysis, multidimensional scaling, or neuronal networks). The 591 inferential articles were subdivided into six categories according to statistical analysis used [35]. If several statistical analyses were reported, we used the result that directly addressed the main research question as stated in the abstract or introduction of the paper, or if there were more research questions presented as equal, the result that was presented first. That is, an article contributed one measure only. In order to check the reliability of the coding, 50 randomly selected articles were independently coded by the second and the third author. Agreement in the lowest, and thus strictest, category of the decision tree was 92%. Cases of disagreement were solved by discussion.

After excluding 9 quantitative reviews, and 51 articles using a statistical method other than categories a-d (see Table 1) from the group of 591 inferential articles, we extracted p-value, SS, and ES for the remaining 531 articles.For 2 articles no SS, and for another 136 articles no ES could be calculated due to missing statistical information (e.g. degrees of freedom) which could not be estimated based on other given information. We also computed exact p-values for all studies that did not indicate an exact p-value based on test statistics and degrees of freedom. This led to the final data set of 395 studies. SS was determined as the total number of participants included for testing the major hypothesis. For the computations *ES: A Computer Program and Manual for Effect Size Calculation* [36] was used. ES for regression analysis were converted according to Peterson and Brown [37]. We corrected all ES as suggested by Thompson [38] (for more details see supporting information S1).

**Table 1:** Correlation between corrected effect size r and sample size for different statistical analysis categories.

Statistical analysis category	k	*r*_ESxN_	95% CI
a) tests for categorical data	41	−.28	[−.54; .03]
b) tests of mean difference and analyses of variance	184	−.52	[−.62; −.40]
c) Correlations/linear regressions	90	−.36	[−.53; −.17]
d) rank order tests	26	−.53	[−.76; −.18]
Total	341	−.45	[−.53; −.36]

*Note*: r_ESxN_ is the bivariate Spearman rank correlation between the corrected effect size *r* and the total sample size (*N*).

*P* values were analyzed by a caliper test, which compares the frequency of values in equal sized intervals just below and just above the threshold value of statistical significance [39], [40]. The basic idea of a caliper test is that if there are considerably more results below than above the critical value of *p = .05*, this value does somehow affect what is published. Although one cannot be sure about the overall distribution of p-values in published literature, a drop exactly at the commonly used p<.05 margin would be very strange. For the caliper test probability values of test statistics were transformed into *z* values. We used the conventional .05 significance level and thus the corresponding two-sided *z* value of 1.96 as the critical value for the caliper test. Seven articles that used a one-sided test statistic were excluded from this analysis as they use a different critical level (z = 1.64) and thus should have been analyzed separately [40].

### Power survey

Since reliance on statistical power can lead to an ES-SS relationship, we contacted the corresponding authors of all 1,000 articles by email and asked them to participate in an online survey. In the survey we described to them that we had randomly selected 1,000 articles from the PsycINFO database to extract the SS and ES, and that one of their papers happened to be selected. Then we asked the authors to estimate the direction and size of the correlation between ES and SS in these papers. Of the 1,000 email addresses 146 produced error replies (false address, retirement, university change, etc.). From the rest, 282 corresponding authors (33%) clicked the link and 214 (25%) answered the question about the correlation.

## Results

### Correlation between Effect Size and Sample Size

**Figure 2:**
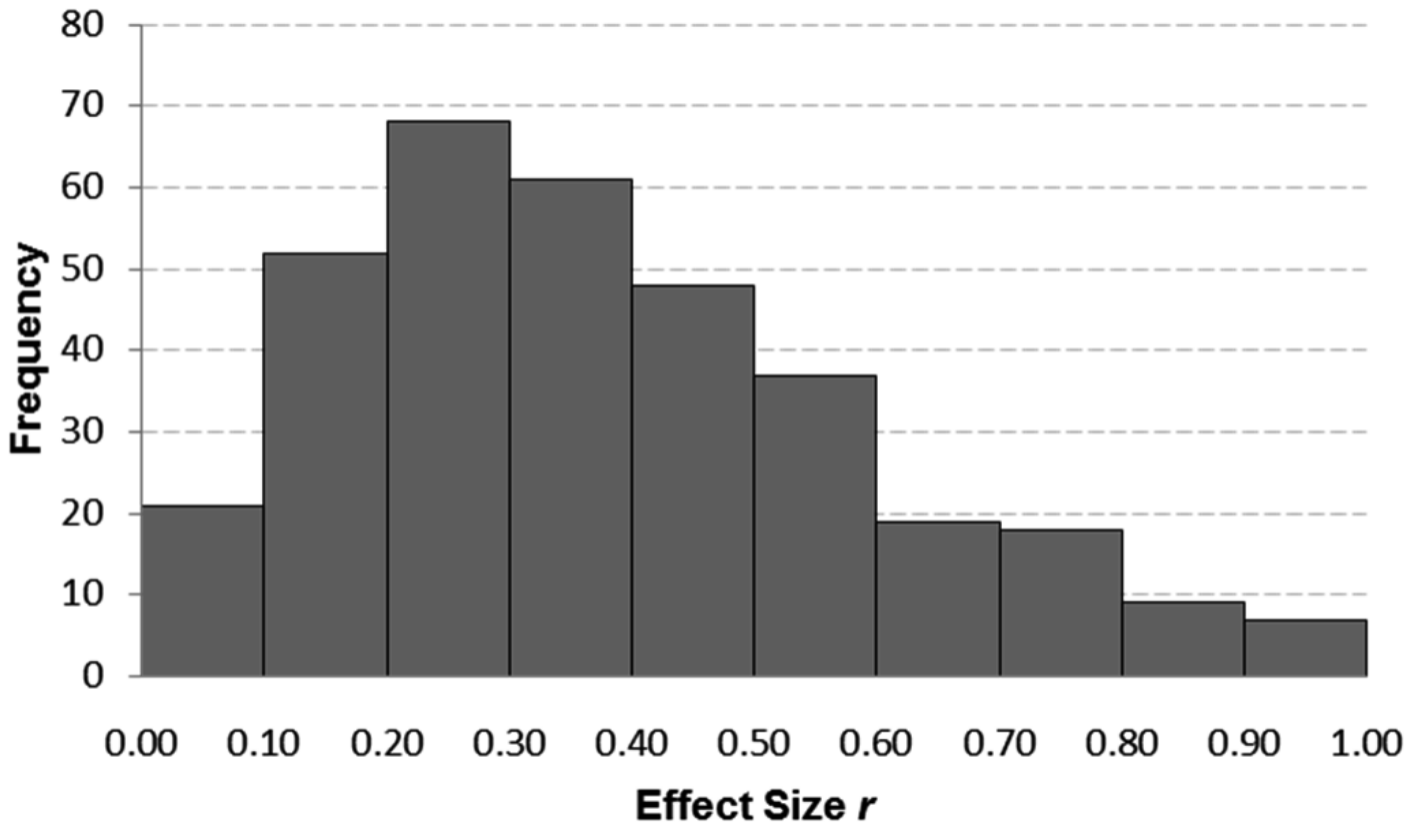
Frequency plot of effect sizes r of all 341 valid studies with sample size between 10 and 1000.

**Figure 3:**
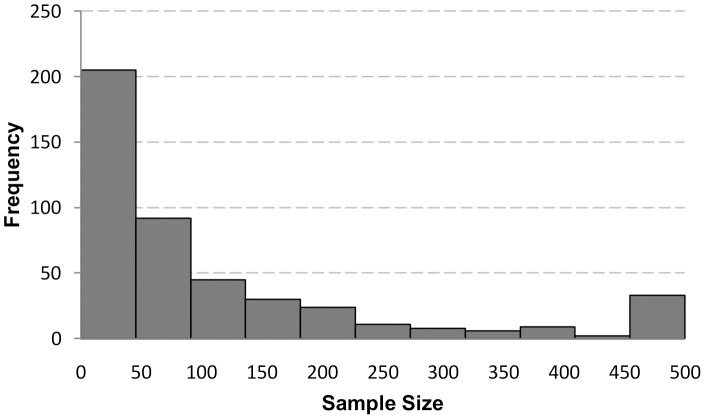
Distribution of sample sizes of all eligible 447 articles. Note: bin from 450 to 500 includes all studies with sample size bigger than 450 but less than 1,000.

**Figure 4:**
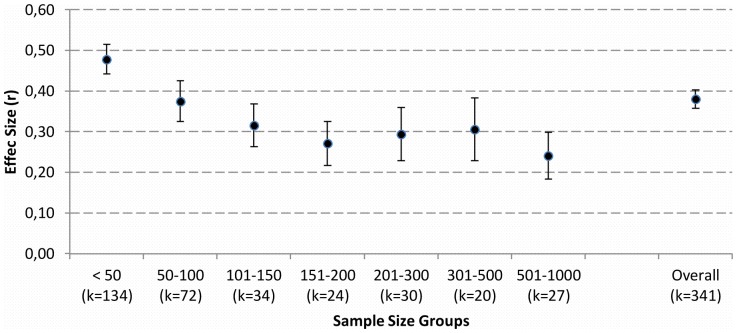
Average effect sizes r for categories of different sample sizes. Note: k indicates the number of articles in each category. Vertical lines indicate the 95% confidence interval for the mean effect sizes.

**Figure 5:**
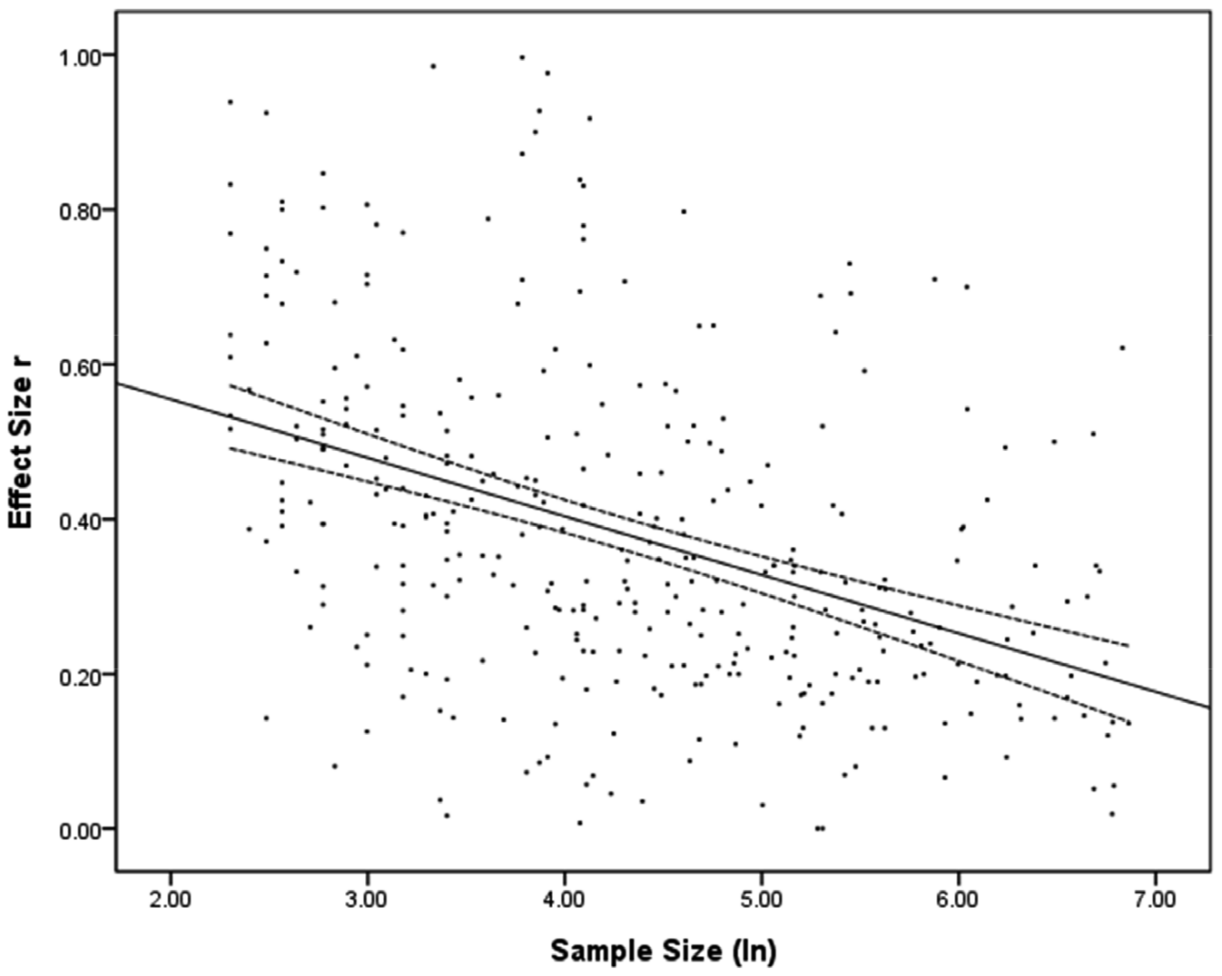
Corrected effect size r plotted against logarithmically transformed sample size. Note: Dashed lines indicate the 95% confidence intervals for the regression line.

The distribution of corrected ES and of SS is given in Figures 2 and 3. Since both distributions showed a positive skew Spearman rank correlations were used to compute the ES-SS relationship. The overall correlation between ES and SS was *r* = −.54 [95% CI: −.60; −.50]. This correlation decreased a bit [*r* = −.45; 95% CI: −.53; −.36] after excluding articles with extreme SS (less than N = 10 and bigger than N = 1000; 54 studies). Figure 4 depicts the ES-SS correlation for categories of different SS, showing that ES-SS correlation was very high (*r* = −.48) for small samples (N<50), whereas this correlation decreased to about *r* = −.30 for bigger samples, and to *r* = −.24 for the category of the largest SS (501<N<1000). Figure 5 depicts this relationship in terms of a linear regression: the logarithmically transformed SS accounted for 18% of the variation in ES (*b* = −.08, 95% CI [−.09; −.06], SE_b_ = .009, *β* = −.42, *R^2^* = .18). Put differently, a decrease of 1 unit in SS (measured in ln, i.e., 2.7 participants, non-transformed) led to an increase of .08 units in ES *r*. Explained in a more illustrative way, in a study with a SS of 50 we would expect an ES that is .05 higher than in a study with a SS of 100, and .19 higher than in a study with the SS of 600.

Table 1 reports the ES-SS correlation for each type of statistical analysis. The relationship varied between *r* = −.28 and *r* = −.53 for the separate categories, with a sizable correlation in all categories. We therefore conclude that the relationship between ES and SS exists independently of the statistical test used.

Finally, we tested whether explicit power considerations were the reason for this correlation. We measured the ES-SS correlation in studies that reported power, or at least mentioned it in the method or discussion section. Only 19 of the 341 studies (5%) computed power, and another 27 (8%) mentioned power. There was no difference in correlation between studies mentioning power and studies failing to do so: all correlations were significant and negative (all *r*<−.35) and the respective confidence intervals were overlapping. To sum up, authors do seldom use power analysis; at least they fail to talk about it explicitly in their papers and the ES-SS correlation in studies of authors who do report power is essentially the same as in the studies of the other authors.

### Results of power survey

The estimated correlations covered the whole range of possible values between −1 and +1. Specifically, 21% of the respondents expected a negative, 42% a positive, and another 37% exactly a zero correlation between ES and SS (mean r = 0.08, SD = 0.02, median r = 0.00). Thus, on average, authors estimated that there will be no ES-SS correlation, rendering power considerations unlikely as the source of a possible ES-SS relationship.

### Distribution of *p* values

**Figure 6:**
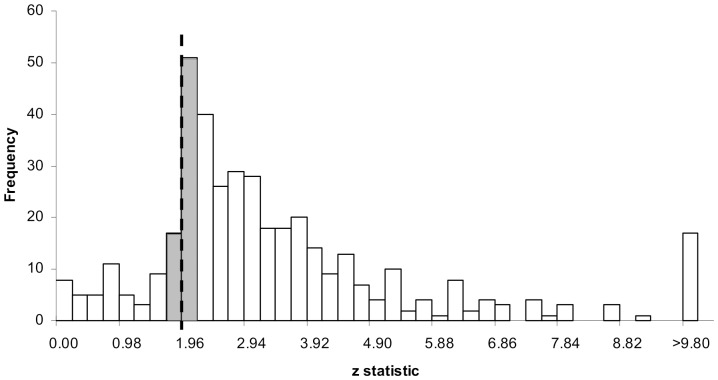
Distribution of z-transformed p values. Note: Dashed line specifies the critical z-statistic (1.96) associated with p = .05 significance level for two-tailed tests. Width of intervals (0.245 i.e. a multiple of 1.96) correspond to a 12.5% caliper.

Figure 6 displays the distribution of *z* scores derived from the reported *p* values. The dashed line specifies the critical *z* value of 1.96 (5% significance level). The shaded bars represent *z* scores that fall just above and just below the threshold for a 12.5% caliper (lower interval: 1.71≤*z*<1.96; upper interval: 1.96≤*z*<2.20). The figure shows a peak in the number of articles with a *z* score just above the critical level, while there are few observations just below it. Table 2 presents the frequencies for different calipers. The ratio of published studies just reaching significance to those just failing is about 3∶1 – a highly unlikely result: the null hypothesis that a *z* score just above and just below an arbitrary value is about equally likely can be rejected for all three calipers.

**Table 2:** Caliper tests of z-values.

	z-interval	over caliper	under caliper	*p* value
10% caliper	1.76; 2.16	15	39	<.001
15% caliper	1.67; 2.25	22	58	<.001
20% caliper	1.57; 2.35	24	77	<.001

## Discussion

We investigated the relationship between ES and SS in a random sample of papers drawn from the full spectrum of psychological research and found a strong negative correlation of *r* = −.54 (*r* = −.45 after excluding extreme sample sizes). That is, studies using small samples report larger effects than studies using large samples. We also analyzed the distribution of *p* values in a caliper test and found about 3 times as many studies just reaching than just failing to reach significance. Finally, neither asking authors directly, nor coding power from papers, indicated that power analysis was consistently used. This pattern of findings allows only one conclusion: there is strong publication bias in psychological research!

### Publication Bias in Psychology

Publication bias, in its most general definition, is the phenomenon that significant results have a better chance of being published, are published earlier, and are published in journals with higher impact factors [34]. Publication bias has been shown in a diverse range of research areas (political science [29], sociology [39], [40], evolutionary biology [42] and also in some areas of psychology [43], [44]) However, most of these prevalence estimates have been based on the analysis of some journal volumes over a specific course of time or specific meta-analyses. Based on these findings one can only argue that publication bias is a problem in a specific area of psychology (or even only in specific journals) and yet no conclusive empirical evidence for a pervasive problem has been provided, although many see pervasive publication bias as the root of many problems in psychology [27]. In contrast, we were investigating and estimating publication bias over the whole field of psychology using a random sample of journal articles. In our sample we also found publication bias for getting published, but our analysis does not allow identification of its source: failure to write up, failure to submit, failure to send out for review, failure to recommend acceptance, or failure to accept a paper. However, as Stern and Simes [45] pointed out, individual factors cannot be assumed to be independent from editorial factors, since previous experiences may have conditioned authors to expect rejection of non-significant studies [20]. Thus, anticipation of biased journal practice may influence the decision to write up and submit a manuscript. It may even influence how researchers conduct studies in the first place. There are many degrees of freedom for conducting studies and analyzing results that increase the probability of getting a significant result [26], and may therefore be used in order to minimize the danger of non-significant results [27]. Researchers then may be tempted to write up and concoct papers around the significant results and send them to journals for publication. This *outcome selection* seems to be widespread practice in psychology [12], which implies a lot of false positive results in the literature and a massive overestimation of ES, especially in meta-analyses. What is often reported as the “mean effect size” of a body of research can, in extreme cases, actually be the mean ES of the tail of the distribution that only consists of overestimations. Consequently, a substantial part of what we think is secure knowledge might actually be (statistical) errors [46]–[49]. Ioannidis [50], for example, analyzed frequently cited clinical studies and their later replications. He found that many studies, especially those of small SS, reported stronger effects than larger subsequent studies, i.e., replication studies found smaller ES than the initial studies. The tendency of effects to fade over time was discussed as the *decline effect* in *Nature* [51]. Most of the reported examples for the decline effect stem from sciences other than psychology, but Fanelli [52] found that results giving support to the research hypothesis increase down the hierarchy of the sciences. In psychological and psychiatric research the odds of reporting a positive result was around 5 times higher than in Astronomy and in Psychology and Psychiatry over 90% of papers reported positive results.

Publication practice needs improvement. Otherwise misestimation of empirical effects will continue and will threaten the credibility of the entire field of psychology [53]. Many solutions have been proposed, all having their specific credits.

One proposal is to apply stringent standards of *statistical power* when planning empirical research. A review on the reporting of sample size calculation in randomized controlled trials in medicine found that 95% of 215 analyzed articles reported sample size calculations [54]. In comparison, only about 3% of psychological articles reported statistical power analyses [8]. However, the usefulness of power analysis is debatable. For example, in medicine a research practice called *sample size samba* emerged as a direct consequence of requiring power analysis. Sample size samba is the retrofitting of a treatment effect worth of detection to the predetermined number of available participants and seems to be fairly common in medicine [55].

Another proposal requires that studies are *registered* prior to their realization. Unpublished studies can then be traced and included in systematic reviews, or at least the amount of publication bias can be estimated. For many clinical trials study registration and reporting of results is required by federal law, and some medical journals require registration of studies in advance [56].

Another proposal is the *open access* movement, which requires that all research must be freely viewable to the public. Related is *data sharing*, which requires authors to share data when requested. Data sharing practices have been found to be somewhat lacking and have been put forward as one reason impeding scientific progress and replication [41]. However, some journals do encourage data sharing, like the journal *Psychological Science* that earns an Open Data badge, printed at the top of an article. Finally, *open-access databases*, where published and unpublished findings are stored, greatly reduce bias due to publication practice (see [57]).

Still another proposal is to install *replication* programs where statistically insignificant results are published as part of the program [58]. However, there is skepticism about the value of replication (see the *special section on behavioral priming and its replication* of *Perspectives on Psychological Science*, Vol. 9, No 1, 2014), and on whether a wide-spread replication attempt will find enough followers in the research community. After all, the payoff from reporting new and surprising findings is larger than the payoff from replication, and replications have a lower chance of being published [59].

#### Emphasizing precision

We favor still another proposal: to *emphasize precision*. Statistical inference is built on three focal characteristics: the *p* value, the ES, and the SS. The most stringent prescription for interpretations exists for the *p*-value: significance interpreted in a dichotomous way regards everything below 5% as irrelevant and useless, and everything above 5% as important and useful. Prescriptions for the interpretation of ES are less strict. It is generally accepted what small, medium, and high ES are, but there is little research on whether this is true empirically [9].

For SS there is no accepted prescription; any SS is acceptable. In consequence, the variance in SS is enormous. In our data set, the smallest sample was N = 1 and the largest was N = 60.599. SS is an index of precision: the bigger the sample, the more precisely can population parameters be estimated. We think that a valid way to improve publication practice is by focusing on the precision of research. More specifically, ES should be supplemented with confidence intervals. The reader can tell from the width of the intervals how accurate, and therefore trustworthy, the estimation is [60]. Studies with large ES, but wide confidence intervals should be interpreted with caution because they probably overestimate the size of the effect.

Confidence intervals as remedy are readily available because the relevant techniques and computer programs do already exist (e.g., Cumming: ESCI [61]). In addition, emphasizing precision does not require that researchers dramatically change their attitudes concerning non-significant findings, but only requires a minor change in editorial policy and reporting practice. With increasing experience researchers will be attentive to the additional information obtained from the confidence interval of the ES and will thus be able to evaluate studies better than by simply relying on *p* values and ES alone.

Note that the information about precision may increase chances of publication primarily for non-significant studies. The size of the confidence interval of the ES indicates how precisely the study managed to measure the underlying effect. A precise measurement may be worth being published, irrespective of whether or not the effect is significant.

### Limitations and Conclusion

In a sample of 1,000 randomly selected papers that appeared in indexed psychological journals of the year 2007, ES was negatively correlated with SS. This indicates that it is the significance of findings which mainly determines whether or not a study is published. Our results stand for the entire discipline of psychology, bearing in mind the following main limitations:

First, we sampled from only one single year of psychological research. It could be that things are changing, to the better or to the worse, and sampling from different years could help decide how reliable our findings are and how dynamic the presumed processes are.

Second, our analysis includes less than half of all papers, namely those which were quantitative and for which we were able to extract data on SS, *p* value, and ES. Those are empirical papers that use mainly classical statistical tests. The whole trade of non-empirical, qualitative, descriptive, and structure detecting research is excluded. The concept of significance has no meaning in most of these excluded areas, and our analysis does not apply to them.

Third, we choose our strategy to focus on the main finding to avoid violating independence. Since authors tend to begin papers with the best data (or develop their argument along the strongest finding), our analysis is not representative for all results within an article. Note, however, that it is the main finding that receives most attention.

Fourth, we generalized over all areas of psychological research. In some areas independence between ES and SS might exist. Our analysis is coarse-grained and the general picture may not apply for all areas.

These limitations should be kept in mind for evaluating how challenging our findings are for psychological research. An extreme interpretation of our findings is that nearly every result obtained in a small sample study overestimates the true effect. This pessimistic view is based on the fact that due to the high ES - SS correlation in conjunction with publication bias mainly overestimated findings tend to make it into publication. However, we opt for a more tempered view: First, probably not all of the small studies will be affected. In addition, there is no problem with large studies: these measure the underlying effect precisely and tend to find significant effects by virtue of high power. Most importantly, Figures 4 and 5 reveal another fact: ES decreases with SS, but does not vanish completely. Rather, studies using large samples still find a considerable ES of about *r* = .25. These large studies deliver a precise estimation of the true ES, which clearly is larger than zero. We thus can conclude with comforting news: Psychology has more to offer than just null effects.

## Supporting Information

Supporting Information S1 **Convertible and computable effect sizes including formula.**
(DOCX)

Supporting Information S2 **Raw Data comprising effect sizes and sample sizes of all empirical quantitative articles.** Note: the complete data set is available on request from the corresponding author.
(SAV)
