# Supplementary material for: Publication Bias in Psychology: A Diagnosis Based on the Correlation between Effect Size and Sample Size
Source: PLoS One. 2014 Sep 5;9(9):e105825. doi: 10.1371/journal.pone.0105825 (PMC4156299; doi:10.1371/journal.pone.0105825)
Supplement: Supporting Information S1 — Convertible and computable effect sizes including formula. (DOCX) [file pone.0105825.s001.docx]

**Supporting Information S1. Convertible and computable effect sizes including formula**

The goal of our survey was to do an overall analysis of effect sizes and therefore we converted all effect sizes into a common metric. Thus, we tried to convert every effect size from the articles into a point-biserial correlation (see Supplementary Table 1 for the number of extracted effect sizes). For further analysis, we corrected the effect sizes by applying the Ezekiel (1930) correction formula as suggested by Thompson (2002) leading to the results found in the paper.

Supplementary Table S1

*Number of convertible and computable (with the program ES) effect sizes among our articles separate for analysis categories including formula used for conversion.*

|  | k | convertible | computable with ES | extraction rate |
| --- | --- | --- | --- | --- |
| *a) Effect sizes for tests for categorical data* | | | | |
| effect size not reported | 49 |  | 32 | 65% |
| odds ratio (OR) ^a^ | 21 | 21 |  | 100% |
| Phi Coefficient (r_φ_) | 4 | 4 |  | 100% |
| Cohen´s d ^b^ | 1 | 1 |  | 100% |
| Total | 75 | 26 | 32 | 77% |
| *b) Effect sizes for tests of mean difference and analyses of variance* | | | | |
| effect size not reported | 251 |  | 153 | 61% |
| (partial) η^2 c^ | 28 | 28 |  | 100% |
| R^2^ ^d^ | 2 | 2 |  | 100% |
| odds ratio (OR) ^a^ | 1 | 1 |  | 100% |
| Cohen´s d ^b^ | 20 | 20 |  | 100% |
| ε | 2 | 0 |  | 0% |
| Total | 304 | 51 | 153 | 67% |
| *c) Effect sizes for correlations and linear regressions* | | | | |
| effect size not reported | 18 |  | 4 | 22% |
| (partial) r | 45 | 45 |  | 100% |
| R^2^ ^d^ | 18 | 18 |  | 100% |
| β ^e^ | 31 | 31 |  | 100% |
| odds ratio (OR)^a^ | 7 | 7 |  | 100% |
| Cohen´s d ^b^ | 1 | 1 |  | 100% |
| arc elasticity | 1 | 0 |  | 0% |
| Total | 121 | 102 | 4 | 88% |
| *d) Effect sizes for rank order tests* | | | | |
| effect size not reported | 29 |  | 25^f^ | 86% |
| Cohen´s d ^b^ | 1 | 1 |  | 100% |
| η^2 c^ | 1 | 1 |  | 100% |
| Total | 31 | 2 | 25 | 87% |

*Notes*:

^a^ Odd ratios were converted by applying the formula by Hasselblad and Hedges (1995), (cf. Lipsey & Wilson, 2001, p. 198; $d=\frac{\left(\ln OR\right)\cdot\sqrt{3}}{\pi}$).

^b^ Formulars for converting between Cohen´s d and point-biserial corrleation (r_pb_) were taken from Lipsey and Wilson (2001, p. 62f; $r_{pb}=\frac{d}{\sqrt{\frac{1}{p\left(1-p\right)}+d^{2}}}$,$d=\frac{r_{pb}}{\sqrt{p\left(1-p\right)\left(1-r_{pb}\right)}}$).

^c^ Formulas for converting between η^2^, f^2^, f, and d were taken from Cohen (1988, pp. 276ff, 281, 284, 285).

^d^ Formulas for converting between R^2^, f^2^, f, and d were taken from Cohen (1988, p. 409ff).

^e^ Standardized regression (β) coefficients were converted according to Peterson and Brown (2005; p. 180, $r=\beta+.05\lambda$).

^f^ Mann-Whitney test scores were converted by applying the formula by Rosenthal (2001), (cf. Field, 2009, p. 550; $r=\frac{Z}{\sqrt{N}}$).

**Supplementary References:**

Cohen, J. (1988). *Statistical Power Analysis for the Behavioral Sciences* (2^nd^ ed.). Hillsdale, NJ: Erlbaum.

Dunlap, W. P. (1999). A program to compute McGraw and Wong's common language effect size indicator. *Behavior Research Methods, Instruments and Computers, 31*, 706–709.

Ezekiel, M. (1930). *Methods of correlational analysis*. New York, NY: Wiley.

Field, A. P. (2009). *Discovering statistics using SPSS* (3^rd^ ed.). Los Angeles, CA: SAGE.

Hasselblad, V., & Hedges, L. V. (1995). Meta-analysis of screening and diagnostic tests. *Psychological Bulletin, 117*, 167-178.

Lipsey, M. W., & Wilson, D. B. (2001). *Practical meta-analysis*. Thousand Oaks, CA: SAGE.

McGraw, K. O., & Wong, S. P. (1992). A common language effect size statistic. *Psychological Bulletin, 111*, 361–365.

Peterson, R. A., & Brown, S. P. (2005). On the use of beta coefficients in meta-analysis. *Journal of Applied Psychology, 90*, 175–181.

Rosenthal, R. (1991). *Meta-analytic procedures for social research* (2^nd^ ed.). New York, NY: SAGE.

Thompson, B. (2002). “Statistical,” “practical,” and “clinical”: How many kinds of significance do counselors need to consider? *Journal of Counseling and Development*, *80*, 64–71.
